# Surgery for degenerative cervical myelopathy in patients with rheumatoid arthritis and ankylosing spondylitis: a nationwide registry-based study with patient-reported outcomes

**DOI:** 10.1007/s00701-022-05382-9

**Published:** 2022-10-15

**Authors:** Siril T. Holmberg, Agnete M. Gulati, Tonje Okkenhaug Johansen, Øyvind O. Salvesen, Vetle Vangen Lønne, Tore K. Solberg, Erling A. Tronvik, Øystein P. Nygaard, Sasha Gulati

**Affiliations:** 1 Department of Neurosurgery, St. Olavs Hospital, 7006 Trondheim, Norway; 2 Department of Neuromedicine and Movement Science, Norwegian University of Science and Technology, Trondheim, Norway; 3 Department of Rheumatology, St. Olavs Hospital, Trondheim, Norway; 4 Department of Public Health and General Practice, Norwegian University of Science and Technology, Trondheim, Norway; 5 Department of Neurosurgery and the Norwegian Registry for Spine Surgery, University Hospital of North Norway, Tromsø, Norway; 6 Institute for Clinical Medicine, The Arctic University of Norway, Tromsø, Norway; 7 Norwegian Advisory Unit On Headaches, St. Olavs University Hospital, Trondheim, Norway; 8 National Advisory Unit On Spinal Surgery, St. Olavs Hospital, Trondheim, Norway

**Keywords:** Cervical spine, Decompressive surgery, Degenerative, Degenerative cervical myelopathy, Spine surgery, Cervical spondylotic myelopathy

## Abstract

**Purpose:**

To compare patient-reported outcomes (PROMs) following surgery for degenerative cervical myelopathy (DCM) among patients with rheumatoid arthritis (RA) or ankylosing spondylitis (AS) versus those without rheumatic diseases.

**Methods:**

Data were obtained from the Norwegian Registry for Spine Surgery. The primary outcome was change in the Neck Disability Index (NDI) at 1 year. Secondary endpoints included the European Myelopathy Score (EMS), quality of life (EuroQoL-5D [EQ-5D]), numeric rating scales (NRS) for headache, neck pain, and arm pain, and complications.

**Results:**

Among 905 participants operated between 2012 and 2018, 35 had RA or AS. There were significant improvements in all PROMs at 1 year and no statistically significant difference between the cohorts in mean change in NDI (− 0.64, 95% CI − 8.1 to 6.8, *P* = .372), EQ-5D (0.10, 95% CI − 0.04 to 0.24, *P* = .168), NRS neck pain (− 0.8, 95% CI − 2.0 to 0.4, *P* = .210), NRS arm pain (− 0.6, 95% CI − 1.9 to 0.7, *P* = .351), and NRS headache (− 0.5, 95% CI − 1.7 to 0.8, *P* = .460).

**Discussion and conclusion:**

Our study adds to the limited available evidence that surgical treatment cannot only arrest further progression of myelopathy but also improve functional status, neurological outcomes, and quality of life in patients with rheumatic disease.

## Introduction

Degenerative cervical myelopathy (DCM) is the most common cause of spinal cord impairment and can cause neurological symptoms including gait disturbances, imbalance, loss of dexterity, poor coordination, pain and stiffness in the neck, pain and numbness in limbs, and autonomic alterations that may cause bowel, urinary, and sexual problems [1, 16]. DCM is typically caused by degenerative changes such as disc herniation, ligament hypertrophy or ossification, and osteophyte formation that may lead to spinal cord compression and dysfunction [16]. There are limited data on the epidemiology of DCM, and exact numbers of prevalence or incidence are lacking. In European studies, the prevalence of surgically treated DCM has been estimated between 1.6 and 4.7 per 100,000 inhabitants [2, 14]. Patients with rheumatoid arthritis (RA) or ankylosing spondylitis (AS) are prone to develop inflammatory and degenerative changes in the cervical spine that may, sometimes in combination with degenerative changes, result in myelopathy [7].

Although there is growing evidence that decompressive surgery can halt disease progression and is associated with meaningful improvement in function, pain, and quality of life [4, 5, 8], the data for patients with coexisting rheumatic conditions are sparse. Whether patients with RA or AS experience similar improvements in patient-reported outcomes (PROMs) following surgery remains unclear, and there is also a concern that they are more prone to complications [7]. While untreated DCM can lead to serious morbidity, management of patients with coexisting RA and AS remains controversial [10].

The aim of this study was to compare the effectiveness and safety of surgery for DCM in patients with RA or AS versus patients without rheumatic disease based on PROMs 1 year after surgery.

## Methods

The Regional Committee for Medical Research Ethics approved the study (2016/840), and all participants provided written informed consent. This study was part of the first author’s master thesis at the Faculty of Medicine, Norwegian University of Science and Technology, Trondheim, Norway.

### Study population

Patients were identified through the Norwegian Registry for Spine Surgery (NORspine), a comprehensive nationwide registry for quality control and research. NORspine provides prospectively collected data on patients undergoing surgery for degenerative spinal disorders, and more than 80% of all cervical spine surgeries are included [8]. Patients were eligible if they had a diagnosis of DCM and underwent decompressive surgery between 2012 and 2018. The number of operated levels, surgical approach, and the use and type of instrumentation were performed at the surgeons’ discretion.

### Outcome measures

The primary outcome was change in the Neck Disability Index (NDI) at 1 year [11]. The NDI summary score ranges from 0 to 100, with lower scores indicating less disability. The minimal clinically important change (MCIC) for NDI is approximately 7.5 points [15, 22].

Secondary outcome measures were changes in DCM severity assessed by the European Myelopathy Score (EMS) [9, 21], numeric rating scale (NRS) range 0 to 10 for headache, neck pain, and arm pain at 1 year [3], and quality of life assessed by EuroQoL-5D (EQ-5D) [17]. For EQ-5D, an index value for health status is generated for each patient. Scores range from − 0.6 to 1, where 1 indicates perfect health. Patients’ perceived effect of surgery was assessed using Global Perceived Effect (GPE) scale, a seven-point scale [12]. Surgeons provided data on perioperative complications, and patients reported complications occurring within 3 months of hospital discharge.

### Data collection

Baseline data were collected on admission for surgery where the patients completed a self-administered questionnaire. Using a standard registration form, surgeons recorded data on diagnosis, comorbidity, American Society of Anesthesiologists (ASA) grade, image findings, and surgical procedure. NORspine distributed self-administered questionnaires to the patients by mail 3 and 12 months after surgery.

### Statistical analysis

Statistical analyses were performed with SPSS version 26 (IBM) and software R version 3.6.3. For statistical comparison tests, we defined the significance level as *P* ≤ 0.05. Changes in EMS, NDI, EQ-5D, and NRS were compared with paired sample *T*-test. Missing data were handled with mixed linear model analyses [20]. Because of the potential for type 1 and type 2 errors due to multiple comparisons and limited sample size, findings for analyses of secondary endpoints should be interpreted as exploratory.

## Results

Among 905 patients included in this study, 35 (4%) were diagnosed with either RA (*n* = 25) or AS (*n* = 10). In total, 697 (77%) participants provided PROMs at 3 months and/or 1 year, with no statistically significant differences between the two cohorts (85.7% vs 76.7%, *P* = 0.21). Baseline characteristics are presented in Table 1. Among patients with RA or AS, a posterior surgical approach was more common (62.9% vs 39.4%), and the patients had longer hospital stays (2.7 vs 1.6 days). ASA grade > 2 was more common in patients with RA or AS (37.1% vs 21.2%, *P* = 0.025).

**Table 1:** Personal characteristics and surgical treatments

Demographics (*n* = 905)	Without rheumatic disease (*n* = 870)	With RA or AS (*n* = 35)	*P* value
No. of patients included	870	35	
Age, year (*SD*)	57.4 (± 12.5)	61.9 (± 0.0)	.806
Female	340/870 (39.1%)	25/35 (71.4%)	< .001
Married or partner	607/862 (70.4%)	20/35 (57.1%)	.093
College education	271/821 (33%)	10/34 (29.4%)	.622
Mean body mass index (*SD*)	27.2 (± 4.7)	27.8 (± 6.1)	.780
Current smoker	310/860 (36%)	9/35 (25.7%)	.211
Obesity, *BMI* ≥ 30	200/848 (23.6%)	11/33 (33.3%)	.198
Prior cervical spine surgery	97/870 (11.1%)	5/35 (14.3%)	.565
Prior surgery in the same level	20/870 (2.3%)	2/35 (5.7%)	.198
Symptoms > 1 year	175/840 (20.8%)	8/34 (23.5%)	.705
ASA grade > 2	177/836 (21.2%)	13/35 (37.1%)	.025
Mean preoperative EMS score (*SD*)	14.4 (± 2.4)	12.4 (± 2.5)	< .001
Mean preoperative NDI (*SD*)	34.5 (± 16.7)	45.9 (± 14.5)	< .001
Mean preoperative EQ-5D (*SD*)	0.45 (± 0.32)	0.28 (± 0.29)	.165
Mean preoperative NRS headache (*SD*)	3.2 (± 3.1)	3.8 (± 2.7)	.011
Mean preoperative NRS neck (*SD*)	4.7 (± 2.9)	6.0 (± 2.6)	.436
Mean preoperative NRS arm (*SD*)	5.0 (± 2.9)	5.5 (± 2.7)	.341
Preoperative diagnostic imaging			
Preoperative MRI—No (%)	850/870 (97.7%)	35/35 (100%)	.364
Preoperative CT—No (%)	109/870 (12.5%)	13/35 (37.1%)	< .001
Surgical approach (%)			
Anterior	525/870 (60.3%)	12/35 (34.3%)	.002
Posterior	343/870 (39.4%)	22/35 (62.9%)	.006
Instrumented fusion	11/870 (1.3%)	6/35 (17.1%)	< .001
Circumferential	2/870 (0.2%)	1/35 (2.9%)	.008
Number of levels > 1	429 (49.3%)	22/35 (62.9%)	.116
Spine level of surgery			
C0/C1	0/870 (0.0%)	3/35 (8.6%)	< .001
C1/C2	3/870 (0.3%)	1/35 (2.9%)	.028
C2/C3	52/870 (6%)	2/35 (5.7%)	.949
C3/C4	243/870 (27.9%)	15/35 (42.9%)	.055
C4/C5	370/870 (42.5%)	19/35 (54.3%)	.168
C5/C6	560/870 (64.4%)	20/35 (57.1%)	.382
C6/C7	315/870 (36.2%)	12/35 (34.3%)	.817
C7/Th1	34/870 (3.9%)	1/35 (2.9%)	.752
Mean operation time in minutes (*SD*)	91.5 (± 41.2)	118.5 (± 66.3)	.003
Postoperative days in hospital no. (*SD*)	1.6 (± 1.73)	2.7 (± 2.21)	< .001

*Abbreviations*: *NDI*, Neck Disability Index; EMS, European Myelopathy Score; *NRS*, numeric rating scale; *ASA*, American Society of Anesthesiologists; *MRI*, magnetic resonance imaging; *CT*, computed tomography

### Primary outcome

PROMs are presented in Table 2. For the total study population, there was a significant improvement in NDI (10.0 points, 95% CI 8.4 to 11.5, *P* < 0.001). Patients with RA or AS reported a higher NDI score before surgery (46.7 vs 34.5 points), but there was no statistically significant difference in mean change between the cohorts at 1 year in the complete case analysis (− 0.64, 95% CI − 8.1 to 6.8, *P* = 0.867). Patients with RA or AS experienced a significantly larger improvement in the mixed model analysis (difference in mean change − 8.8 points, 95% CI − 13.8 to − 3.7, *P* < 0.001). The change in NDI exceeded the MCIC of 7.5 points for both cohorts.

**Table 2:** Outcomes at 1 year in patients operated for degenerative cervical myelopathy

Patients without rheumatic disease				Patients with RA or AS				*P* value
	Baseline, mean (*SD*)	One year, mean (*SD*)	Mean difference (95% CI)	Baseline, mean (*SD*)	One year, mean (*SD*)	Mean difference (95% CI)	Difference in mean change between groups (95% CI)	
Outcome variable (complete case analysis)								
NDI	34.5 (± 16.7)	24.5 (± 17.9)	9.9 (8.4 to 11.5)	46.8 (± 13.9)	36.2 (± 14.0)	10.6 (4.4 to 16.8)	− 0.64 (− 8.1 to 6.8)	.867
EQ-5D	0.46 (0.32)	0.62 (0.30)	− 0.15 (− 0.19 to − 0.12)	0.31 (0.28)	0.57 (0.25)	− 0.25 (− 0.41 to − 0.10)	0.10 (− 0.04 to 0.24)	.168
EMS	14.4 (± 2.4)	15.3 (± 2.3)	− 0.88 (− 1.1 to − 0.7)	12.1 (± 2.0)	14.2 (± 2.2)	− 2.2 (− 1.0 to − 3.8)	1.3 (0.4 to 2.2)	.003
NRS neck pain	4.7 (3.0)	3.0 (2.8)	1.7 (1.4 to 2.0)	5.8 (2.7)	3.3 (2.2)	2.5 (3.6 to 4.7)	− 0.8 (− 2.0 to 0.4)	.210
NRS arm pain	5.1 (2.9)	3.5 (2.8)	1.6 (1.9 to 10.5)	5.6 (2.6)	3.3 (2.5)	2.3 (0.8 to 3.3)	− 0.6 (− 1.9 to 0.7)	.351
NRS headache	3.3 (3.1)	2.2 (2.6)	1.0 (0.77 to 1.3)	3.7 (2.9)	2.2 (1.9)	1.5 (0.3 to 2.5)	− 0.5 (− 1.7 to 0.8)	.460
Outcome variable (mixed linear model analysis)								
NDI	34.7 (17.7)	25.1 (22.2)	9.5 (8.0 to 11.0)	44.6 (16.3)	32.8 (18.4)	11.9 (5.2 to 18.6)	− 8.8 (− 13.8 to − 3.7)	.001
EQ-5D	0.45 (0.32)	0.61 (0.37)	− 0.16 (− 0.19 to − 0.13)	0.28 (0.29)	0.57 (0.29)	− 0.28 (− 0.43 to − 0.14)	0.10 (0.10 to 0.19)	.017
EMS	14.4 (2.4)	15.3 (2.7)	− 0.8 (− 1.0 to − 0.7)	12.3 (2.6)	14.3 (2.8)	− 2.0 (− 2.8 to − 1.2)	1.53 (0.9 to 2.2)	.001
NRS neck pain	4.7 (3.0)	3.0 (3.6)	1.7 (1.5 to 2.0)	6.0 (2.7)	3.3 (2.5)	2.7 (1.7 to 3.7)	− 0.72 (0.4 to − 1.5)	.073
NRS arm pain	5.0 (3.0)	3.4 (3.8)	1.6 (1.4 to 1.9)	5.5 (2.7)	3.3 (2.9)	2.2 (0.8 to 3.4)	− 0.15 (0.4 to − 0.9)	.714
NRS headache	3.2 (3.2)	2.1 (3.6)	1.0 (0.8 to 1.3)	3.7 (2.8)	2.2 (2.3)	1.6 (0.6 to 2.6)	− 0.27 (− 1.05 to 0.52)	0.500

*Abbreviations*: *NDI*, Neck Disability Index; *EMS*, European Myelopathy Score; *NRS*, numeric rating scale

### Secondary outcomes

There were significant improvements in all PROMs at 1 year for both cohorts. Complete case analyses showed no statistically significant difference between the cohorts in mean change in EQ-5D (0.10, 95% CI − 0.04 to 0.24, *P* = 0.168), neck pain NRS (− 0.8, 95% CI − 2.0 to 0.4, *P* = 0.210), arm pain NRS (− 0.6, 95% CI − 1.9 to 0.7, *P* = 0.351), and headache NRS (− 0.5, 95% CI − 1.7 to 0.8, *P* = 0.460). In the mixed model analysis, patients with RA or AS experienced statistically significant larger improvement in EQ-5D compared to those without rheumatic disease (mean difference − 0.28, 95% CI − 0.43 to − 0.14, *P* < 0.001). Patients with RA or AS had lower EMS scores at both baseline and at 1 year compared to patients without rheumatic disease. Improvement in EMS was larger in patients with RA or AS compared to those without. The change in EQ-5D for the total study population represents a moderate clinical change with an effect size of 0.51.

**Fig. 1:**
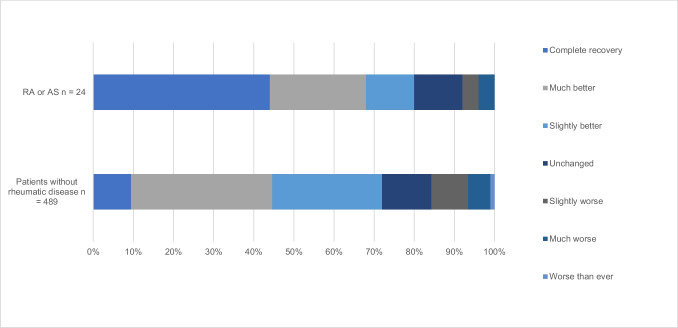
Patients’ perceived benefit of surgery for DCM after 1 year

Patients’ perceived benefit of surgery assessed by the GPE is presented in Fig. 1. There was no statistically significant difference between the groups in the proportion of patients reporting “complete recovery” or feeling “much better” (25.1% vs 31.4%, *P* = 0.395).

Details of surgical treatment and complications are presented in Table 3. Patients with RA or AS were more likely to experience complications and adverse events within 3 months (42.9% vs 26.9%). There were no statistically significant differences between the two groups in perioperative complications.

**Table 3:** Surgeon and patient-reported complications

Variable	Patients without rheumatic disease	Patients with RA or AS	*P* value
Patients with complications, no (%)	234/870 (26.9%)	15/35 (42.9%)	0.005
Perioperative complications, no. (%)	13/870 (1.5%)	0/35 (0.0%)	0.466
Unintentional durotomy	4 (0.5%)	0 (0.0%)	
Iatrogenic spinal cord injury	2 (0.2%)	0 (0.0%)	
Wrong level surgery	0 (0.0%)	0 (0.0%)	
Misplacement of implant	0 (0.0%)	0 (0.0%)	
Perioperative bleeding/postoperative hematoma	2 (0.2%)	0 (0.0%)	
Esophageal injury	0 (0.0%)	0 (0.0%)	
Major blood vessel injury	0 (0.0%)	0 (0.0%)	
Cardiovascular complications	1 (0.1%)	0 (0.0%)	
Respiratory complications	1 (0.1%)	0 (0.0%)	
Anaphylactic reaction	0 (0.0%)	0 (0.0%)	
Other complications	5 (0.6%)	0 (0.0%)	
Patient-reported complications within 3 months, no. (%)	227/656 (34.6%)	15/30 (50.0%)	0.014
Deep wound infection	8 (1.2%)	1 (3.3%)	
Superficial wound infection	32 (4.9%)	3 (10%)	
Urinary tract infections	38 (5.8%)	3 (10%)	
Pneumonia	12 (1.8%)	0 (0.0%)	
Deep venous thrombosis	7 (1.1%)	0 (0.0%)	
Pulmonary embolism	5 (0.8%)	0 (0.0%)	
Dysphagia	69 (10.5%)	3 (10%)	
Dysphonia	57 (8.7%)	5 (16.7%)	

## Discussion

Although patients with RA or AS had more complications, they experienced improvements in their conditions after surgery for DCM that were similar to those of the patients without rheumatic disease. Our study adds to the limited available evidence that surgical treatment cannot only arrest further progression of myelopathy but also improve functional status, neurological outcomes, and quality of life in patients with rheumatic disease [10].

Patients with RA or AS reported higher disability and more severe myelopathy before surgery. Possible explanations include delay in diagnosis, disability due to the rheumatic disease itself, differences in disease progression and pathology, additional comorbidity, and a higher threshold for surgery in patients with RA or AS. In our study, the proportion of patients with symptoms exceeding 1 year was similar for both cohorts. A posterior surgical approach and instrumented fusion were more common in patients with RA or AS, suggestive of more pronounced DCM and multilevel involvement. A recent trial showed similar outcomes following anterior and posterior surgical approaches, but with higher complication rates for the former mainly due to more postoperative dysphagia and dysphonia [6]. There have been concerns regarding safety profile of surgery in patients with RA or AS due to medical treatments which may affect surgical outcomes and increase the risk of complications [7]. In our study, patients with RA or AS had an increased risk of complications after surgery, and this should be clearly communicated to patients prior to surgery. Life-threatening complications and early reoperations were fortunately rare.

Timely diagnosis of rheumatic disease and adequate medical treatment are likely to reduce the risk of developing DCM requiring surgery; however, the cases that need surgery are becoming more complex [10]. While conservative therapy can alleviate pain, surgery might be necessary to prevent serious morbidity [10, 13, 19]. As residual symptoms are common following surgery, early MRI and prompt referral to a spine specialist should be a priority in patients with clinical manifestations suggestive of myelopathy.

### Limitations

Lack of verification of RA and AS diagnoses according to validated disease criteria by a rheumatologist is an important limitation. The two cohorts of patients were unequal relative to the number of participants, and as perhaps expected were not balanced for all baseline and treatment factors. Furthermore, the pathophysiology of DCM in patients with rheumatic disease is likely to differ from those without. Loss to follow-up is a concern. However, a previous study from NORspine showed no difference in outcomes between responders and non-responders [18]. NORspine only includes patients that actually undergo surgery, and unfortunately, we do not have any information about patients ineligible for surgical treatment due to, for example, frailty, comorbidity, and lack of motivation to undergo surgery. Patient characteristics, indications, and surgical strategies may vary between institutions and countries, and results from our study might differ from other countries and clinical settings. Another limitation is that patients in the cohort with rheumatic disease are carefully selected for surgery and might not be representative of the total population of patients with degenerative cervical myelopathy and RA or AS. Moreover, the relatively low number of patients with RA or AS is also a limitation and may impact the generalizability of the results.

## Conclusion

Patients with RA or AS experienced similar improvement following surgery for DCM compared to patients without rheumatic disease at the expense of more complications. Surgical treatment cannot only arrest further progression of DCM in patients with RA or AS, but also improve functional status, neurological outcomes, and quality of life.

## Data Availability

No additional data available.
